# Magnetism and ion diffusion in honeycomb layered oxide ${\hbox {K}_2\hbox {Ni}_2\hbox {TeO}_6}$

**DOI:** 10.1038/s41598-020-75251-x

**Published:** 2020-10-27

**Authors:** Nami Matsubara, Elisabetta Nocerino, Ola Kenji Forslund, Anton Zubayer, Konstantinos Papadopoulos, Daniel Andreica, Jun Sugiyama, Rasmus Palm, Zurab Guguchia, Stephen P. Cottrell, Takashi Kamiyama, Takashi Saito, Alexei Kalaboukhov, Yasmine Sassa, Titus Masese, Martin Månsson

**Affiliations:** 1 Department of Applied Physics, KTH Royal Institute of Technology, 10691 Stockholm, Sweden; 2 Department of Physics, Chalmers University of Technology, 41296 Göteborg, Sweden; 3 Faculty of Physics, Babes-Bolyai University, 400084 Cluj-Napoca, Romania; 4 Neutron Science and Technology Center, Comprehensive Research Organization for Science and Society (CROSS), Tokai, Ibaraki 319-1106 Japan; 5 Laboratory for Muon Spin Spectroscopy, Paul Scherrer Institute, 5232 Villigen, PSI Switzerland; 6 ISIS Muon Facility, Rutherford Appleton Laboratory, Didcot, Oxfordshire OX11 0QX UK; 7 Institute of Materials Structure Science, High Energy Accelerator Research Organization, 203-1 Shirakata, Tokai, Ibaraki 319-1106 Japan; 8 Microtechnology and Nanoscience, Chalmers University of Technology, 41296 Göteborg, Sweden; 9 Department of Energy and Environment, Research Institute of Electrochemical Energy (RIECEN), National Institute of Advanced Industrial Science and Technology (AIST), Ikeda, Osaka 563-8577 Japan; 10 AIST-Kyoto University Chemical Energy Materials Open Innovation Laboratory (ChEM-OIL), National Institute of Advanced Industrial Science and Technology (AIST), Sakyo-ku, Kyoto, 606-8501 Japan

**Keywords:** Chemistry, Energy science and technology, Materials science, Physics

## Abstract

In the quest for developing novel and efficient batteries, a great interest has been raised for sustainable K-based honeycomb layer oxide materials, both for their application in energy devices as well as for their fundamental material properties. A key issue in the realization of efficient batteries based on such compounds, is to understand the K-ion diffusion mechanism. However, investigation of potassium-ion (K$^+$) dynamics in materials using e.g. NMR and related techniques has so far been very challenging, due to its inherently weak nuclear magnetic moment, in contrast to other alkali ions such as lithium and sodium. Spin-polarised muons, having a high gyromagnetic ratio, make the muon spin rotation and relaxation ($\mu ^+$SR) technique ideal for probing ions dynamics in these types of energy materials. Here we present a study of the low-temperature magnetic properties as well as K$^+$ dynamics in honeycomb layered oxide material ${\hbox {K}_2\hbox {Ni}_2\hbox {TeO}_6}$ using mainly the $\mu ^+$SR technique. Our low-temperature $\mu ^+$SR results together with complementary magnetic susceptibility measurements find an antiferromagnetic transition at $T_{\mathrm{N}}\approx 27$ K. Further ${\mu}^{+}$SR studies performed at higher temperatures reveal that potassium ions (K$^+$) become mobile above 200 K and the activation energy for the diffusion process is obtained as $E_{\mathrm{a}}=121 (13)$ meV. This is the first time that K$^+$ dynamics in potassium-based battery materials has been measured using $\mu ^+$SR. Assisted by high-resolution neutron diffraction, the temperature dependence of the K-ion self diffusion constant is also extracted. Finally our results also reveal that K-ion diffusion occurs predominantly at the surface of the powder particles. This opens future possibilities for potentially improving ion diffusion as well as K-ion battery device performance using nano-structuring and surface coatings of the particles.

## Introduction

**Figure 1:**
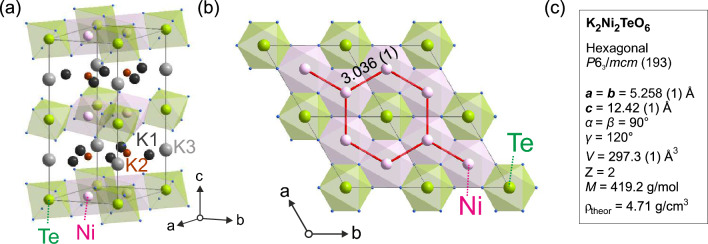
(**a**) Honeycomb layered structure of ${\hbox {K}_2\hbox {Ni}_2\hbox {TeO}_6}$ showing the Ni atoms (pink), Te atoms (green), O atoms (blue), along with the K atoms occupying different crystallographic sites [K1 (black), K2 (red), and K3 (gray)]. The layered structure allow the K ions to become mobile in a two-dimensional (2D) fashion, as reported in Ref.^1^. (**b**) View along the c-axis showing the honeycomb structure. Red lines indicate Ni–Ni network (with interatomic distance in Å). (**c**) The crystal structure information of ${\hbox {K}_2\hbox {Ni}_2\hbox {TeO}_6}$ is extracted from room temperature-NPD data (Fig. 2a,b) *Z* is the number of formula units in the unit cell. Figures of crystal structure were created by Diamond version 4.6.3.

Layered oxides have attracted considerable attention over the last decades owing to their intriguing physical and chemical properties across a wide scope of science, including, phase transitions (e.g. antiferromagnetism, superconductivity, Kitaev magnet)^2–5^, thermodynamics (e.g. fast ionic conductivity)^6^ and unusual electromagnetic spin interactions (multiferroics, high-voltage electrochemistry)^7–9^. Layered oxides consisting of alkali atoms sandwiched between slabs with transition metal atoms (commonly referred to as layered transition metal oxides, TMOs), have been extensively investigated. This is especially true for TMOs adopting the chemical composition $AM\hbox {O}_2$, where *A* denotes an alkali atom and *M* is typically a transition metal atom. Such compounds have raised interests not only from a fundamental point of view but also for applications. For instance, $\hbox {Na}_{\mathrm{x}}\hbox {CoO}_2$ is a widely known material exhibiting a rich phase diagram containing intriguing physical properties at low-temperature,s such as spin density waves^10^, superconductivity (hydrated compound^11,12^), metal-insulator transitions^13^, and in addition also unique magnetic and charge ordering phases^14^.

In electrochemistry, $\hbox {Na}_{\mathrm{x}}\hbox {CoO}_2$ has also been investigated as a cathode material in Na-ion batteries, not only from environmental (sustainability) point of view, but also for its fast sodium-ion diffusive capabilities^15–17^. Despite this, another class of layered oxides has emerged to supersede $\hbox {Na}_{\mathrm{x}}\hbox {CoO}_2$, such as ${\hbox {Na}_2\hbox {Ni}_2\hbox {TeO}_6}$ (or equivalently as ${\hbox {Na}_{2/3}\hbox {Ni}_{2/3}\hbox {Te}_{1/3}\hbox {O}_2}$)^18,19^ and ${\hbox {K}_2\hbox {Ni}_2\hbox {TeO}_6}$ (${\hbox {K}_{2/3}\hbox {Ni}_{2/3}\hbox {Te}_{1/3}\hbox {O}_2}$)^1^, which show higher voltage (vs Na/Na$^+$ ; K/K$^+$) cation electrochemistry and better structural stability.

${\hbox {K}_2\hbox {Ni}_2\hbox {TeO}_6}$ adopts essentially the same crystal structure as ${\hbox {Na}_2\hbox {Ni}_2\hbox {TeO}_6}$, but with a significant increase of the interslab distance owing to the larger potassium atoms. The K-ion layers reside in between slabs consisting of Ni octahedra with surrounding Te octahedra creating a honeycomb structure (see Fig. 1a,b). In addition to its application for rechargeable battery devices, interesting low-temperature magnetic properties are anticipated in ${\hbox {K}_2\hbox {Ni}_2\hbox {TeO}_6}$, arising from the regular honeycomb configuration of Ni atoms. Such structure is in itself not geometrically frustrated, however, the interplay between antiferromagnetic (AFM) interactions, anisotropies and bond-dependent interactions, can trigger exotic magnetic states^20^. Moreover, complex magnetic structures can be expected^4,19^ owing to the competition between the direct interactions of magnetic Ni atoms and exchange interactions through the non-magnetic atoms. Finally, the large ionic radii of potassium cations with resulting increase in the interslab distance, influences not only the electronic and spin interactions but also the K diffusion mechanism and properties.

In contrast to Na and Li, K has a weak nuclear magnetic moment that makes this interaction difficult to probe. This places muon spin rotation and relaxation ($\mu ^+$SR) measurements at the frontier of techniques for probing both static and dynamic properties of K-ion nuclear spin. This comes from the unique properties of muons that has a charge, a high gyromagnetic ratio and an appropriate lifetime. In particular for oxide materials, the positive muon is typically strongly bound to the negatively charged oxygen atoms at a distance of 1 Å, and interact with both nuclear and electronic moments in the matter. This means that the muon itself remain static and may couple to as well as *sense* even the weak nuclear moment of K, given the high gyromagnetic ratio of the muon. In addition, $\mu ^+$SR is simultaneously ideal to study both long-range and short-range static magnetic order as well as electronic spin dynamics. Here we report the first measurements of magnetic properties as well as K-ion dynamics in honeycomb layered ${\hbox {K}_2\hbox {Ni}_2\hbox {TeO}_6}$ oxide material using $\mu ^+$SR. Room-temperature x-ray and neutron powder diffraction experiments confirm that the average crystal structure is in agreement with the reported one^1^. Our studies of low-temperature magnetism in ${\hbox {K}_2\hbox {Ni}_2\hbox {TeO}_6}$ reveal that this material exhibits an AFM transition at $T_{\mathrm{N}}\approx 27$ K and ZF-$\mu ^+$SR oscillation signal suggests commensurate spin ordering down to 2 K. $\mu ^+$SR studies performed on ${\hbox {K}_2\hbox {Ni}_2\hbox {TeO}_6}$ at higher temperatures reveal that potassium ions (K$^+$) are dynamic above 200 K (with an activation energy $E_{\mathrm{a}}=121$ (13) meV extracted from the experimental data), revealing for the first time that K$^+$ dynamics can be measured using $\mu ^+$SR.

## Results

### Room temperature diffraction

**Figure 2:**
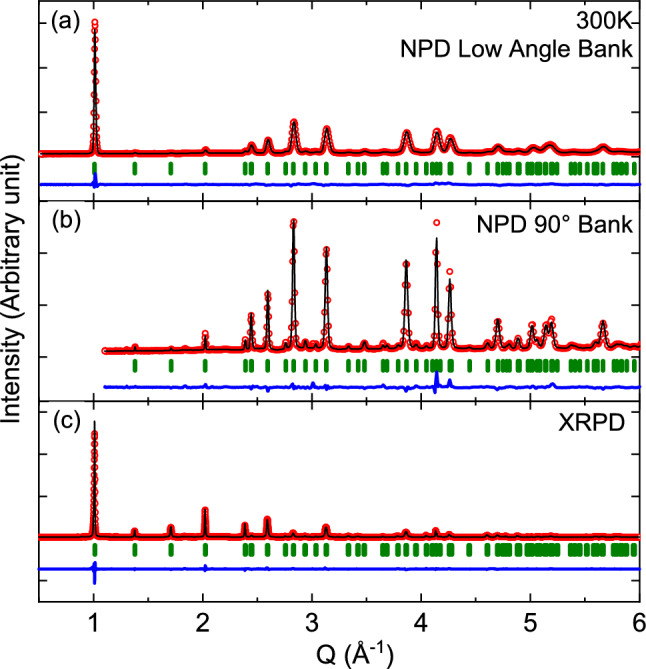
Neutron powder diffraction (NPD) and X-ray powder diffraction (XRPD) results of ${\hbox {K}_2\hbox {Ni}_2\hbox {TeO}_6}$ at $T=300$ K, with corresponding fits and Rietveld refinements for (**a**) NPD low angle detector bank, (**b**) NPD 90$^{\circ }$ detector bank and (**c**) in-house XRPD. Data is shown as open red circles and the fits/refinements as solid black lines. Below the data, green vertical lines mark positions of the allowed Bragg peaks and solid blue line is the difference between the refinement and the data.

The crystal structure of ${\hbox {K}_2\hbox {Ni}_2\hbox {TeO}_6}$ at room temperature ($T=300$ K) was obtained by refinements of both x-ray powder diffraction (XRPD) and neutron powder diffraction (NPD) data. The structural refinement of ${\hbox {K}_2\hbox {Ni}_2\hbox {TeO}_6}$ started from the reported unit cell ($P6_3/mcm$ with *a* = 5.26 Å, *c* = 12.47 Å) and atomic coordinates^1^. The Rietveld fits of high-resolution neutron powder diffraction patterns was challenging due to a significant broadening observed for [*h*, *k*, *l* $\ne$ 0] peaks. Similar broadening profile was reported for ${\hbox {Na}_2\hbox {Ni}_2\hbox {TeO}_6}$, where Karna et al. suggested to introduce an anisotropic strain to improve the crystal structure refinement process^4^. In order to fit both XRPD and NPD data, we used the anisotropic strains based on a spherical harmonics modelling of the Bragg peak broadening using the Fullprof suite. As Karna et al. pointed out in the study of ${\hbox {Na}_2\hbox {Ni}_2\hbox {TeO}_6}$, this strong broadening is probably originated from both the anisotropic displacement of oxygen atoms under thermal fluctuation and the potential alkali-ion distribution due to the weaker interlayer interaction in this type of structure. The average model of the crystal structure provides reasonable fits of both XRPD and NPD data, as shown in Fig. 2. The detailed refinement of the data and the corresponding structure obtained from the NPD data are displayed in Fig. 1 and supplementary materials (Supplementary Tables S1, S2). The average structure is consistent with the previous report^1^. Note that the detailed crystal structure determination is beyond the scope of this paper and the obtained average structure model of ${\hbox {K}_2\hbox {Ni}_2\hbox {TeO}_6}$ is here used for the estimation of the K-ion diffusion coefficient as detailed below in “High-temperature K-ion diffusive properties”. Finally, both XRPD and NPD data reveal that the samples are of very high purity with an absence of impurity phases within the detectable limits of such methods.

### Magnetic susceptibility

**Figure 3:**
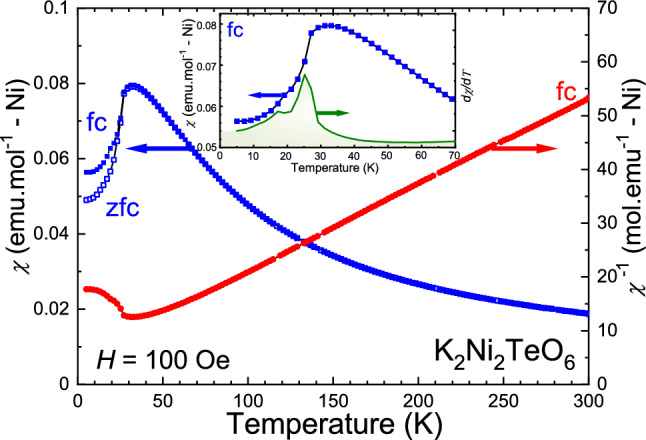
Magnetic susceptibility ($\chi$ (*T*) and 1/$\chi$(*T*)) curves of ${\hbox {K}_2\hbox {Ni}_2\hbox {TeO}_6}$ recorded (upon warming) in zero-field-cooled (*zfc*) and field-cooled (*fc*) modes under an applied magnetic field of 100 Oe, with the corresponding Curie–Weiss fitting as a dotted line. Inset shows the magnified image of the susceptibility plot and of the corresponding differential susceptibility [$d\chi$/*dT*](*T*) curve (green solid line) indicating $T_{\mathrm{N}}$ = 27 K.

Figure 3 displays the DC magnetic susceptibility of ${\hbox {K}_2\hbox {Ni}_2\hbox {TeO}_6}$ measured under a magnetic field of 100 Oe in the temperature range *T* = 5–300 K recorded upon warming the sample. ${\hbox {K}_2\hbox {Ni}_2\hbox {TeO}_6}$ exhibits AFM behaviour with a maximum of the $\chi$ curve at around 33 K. The magnetic transition is made even more evident in the differential susceptibility [$d \chi$/*dT*](*T*) curves, revealing the AFM Néel temperature $T_{\mathrm{N}}\approx 27$ K in both zero-field-cooled (*zfc*) and field-cooled (*fc*) protocols (only *fc* is shown in inset of Fig. 3).

No significant divergence between *zfc* and *fc* magnetisation curves is observed down to the transition temperature.There are slight different between *zfc* and *fc* below the transition probably due to either small ferromagnetic components and/or a partial magnetic disorder. The partial magnetic disorder is often observed in the honeycomb system, owing to the frustration of magnetic spins. Detail will be discussed in the following section. The susceptibility data (1/$\chi$) were fitted with a Curie–Weiss law (using data points above 80 K), yielding a Weiss temperature $\theta_{\mathrm{CW}} = -30.3$ K. The negative Weiss temperature indicates AFM interactions, which could arise from the superexchange interactions between the nearest and the next-nearest neighbours of the Ni layers. Further, an effective magnetic moment, $\mu _{\mathrm{eff}}$ = 2.53 $\mu _{\mathrm{B}}$/Ni was obtained, which is in good agreement with the theoretical spin only value for $\mathrm{Ni}^{2+}$ (2.83 $\mu _{\mathrm{B}}$).

### Low-temperature wTF $\mu ^+$SR  measurements

**Figure 4:**
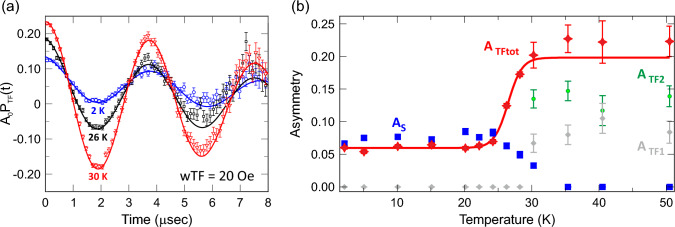
(**a**) $\mu ^+$SR time spectra measured at temperatures $T=$ 2, 26 and 30 K under a weak-transverse field (wTF $=20$ Oe) with the corresponding fits using Eq. (1) (solid lines). For clarity, $A_0$ is the initial asymmetry and $P_{\mathrm{TF}}(t)$ is the muon spin polarisation function. (**b**) Asymmetry plots as functions of temperature, where $A_{\mathrm{TF1}}$, $A_{\mathrm{TF2}}$ and $A_{\mathrm{S}}$ are the initial asymmetries of the related polarisation components. Here the total wTF asymmetry is expressed as $A_{\mathrm{TFtot}}=A_{\mathrm{TF1}}+A_{\mathrm{TF2}}$, which corresponds to the paramagnetic volume fraction of the sample. The sigmoid fit (red solid line) to the $A_{\mathrm{TFtot}}(T)$ data yield the antiferromagnetic transition temperature $T_{\mathrm{N}}\approx 27.1$ K. Data was recorded using the GPS instrument at PSI.

Figure 4a shows the wTF $\mu ^+$SR-time spectra recorded with $H=20$ Oe for three selected temperatures. Here, wTF means that the field is perpendicular to the initial muon spin polarization and its magnitude is very small compared with the internal magnetic field ($H_{\mathrm{int}}$) generated by magnetic spin order and/or disorder. When the temperature decreases below 30 K, the oscillation amplitude of the applied wTF decreases, indicating the appearance of additional internal magnetic fields (i.e. static magnetic order),which rapidly depolarises the muon spin. Below 30 K, the wTF $\mu ^+$SR time spectrum was consequently fitted using a combination of an exponentially relaxing precessing component and a slow-exponentially relaxing non-oscillatory component. The first component comes from the muons stopping in paramagnetic phases, where the internal magnetic field is equivalent to wTF = 20 Oe. The second component corresponds to the magnetically ordered phase, where $H_{\mathrm{int}}$>> wTF. From about 30 K on the other hand, the wTF spectrum was fitted using two exponentially relaxing oscillating components. The two oscillating components stem from two muon sites posing two different internal field distribution widths. The presence of two sites with distinct field distributions is further discussed below. The resulting fit function for the wTF spectra in the wide temperature range across $T_{\mathrm{N}}$ is as follows:

1 $$\begin{aligned} A_0 \, P_{\mathrm{TF}}(t)= & \,{} A_{\mathrm{TF1}}\cos (2\pi f_{\mathrm{TF1}}t + \phi _{\mathrm{TF1}})\times {}\exp {(-\lambda _{\mathrm{TF1}} t)}\nonumber \\&+ A_{\mathrm{TF2}}\cos (2\pi f_{\mathrm{TF2}}t + \phi _{\mathrm{TF2}})\times {}\exp {(-\lambda _{\mathrm{TF2}} t)}\nonumber \\&+ A_{\mathrm{S}}\times {}\exp {(-\lambda _{\mathrm{S}} t)} \end{aligned}$$

where $A_S$ = 0 at *T* $\ge$ $T_{\mathrm{N}}$  and $A_{\mathrm{TF2}}=0$ below $T_{\mathrm{N}}$. $P_{\mathrm{TF}}(t)$ is the muon spin polarisation function, $A_0$ is the initial asymmetry, $A_{\mathrm{TF1}}$, $A_{\mathrm{S}}$ and $A_{\mathrm{TF2}}$ are the asymmetries of the related polarisation components, $2\pi f_{\mathrm{TF1}}$ and $2\pi f_{\mathrm{TF2}}$ are the angular frequency of the Larmor precession under the applied wTF, $\lambda _{\mathrm{TF1}}$, $\lambda _{\mathrm{S}}$ and $\lambda _{\mathrm{TF2}}$ are the exponential relaxation rates for the three components and $\phi _{\mathrm{TF1}}$ and $\phi _{\mathrm{TF2}}$ are the initial phase of the processing signals.

The fitting was performed by setting $\phi _{\mathrm{TF1}}=\phi _{\mathrm{TF2}}$ since the phase should not be muon site dependent. Under such fitting configuration, the obtained asymmetry components are displayed in Fig. 4b. The magnetic transition temperature is obtained from the $A_{\mathrm{TF1}}+A_{\mathrm{TF2}}=A_{\mathrm{TFtot}}$(*T*) curve, because $A_{\mathrm{TFtot}}$ corresponds to the paramagnetic (PM) fraction of the sample. Thus, a step-like change in the $A_{\mathrm{TFtot}}$(*T*) curve around 27 K indicates a transition from a low-temperature magnetically ordered state to a high-temperature PM state. As shown in Fig. 4b, temperature dependence of $A_{\mathrm{TFtot}}$(*T*) has been fitted with a sigmoid function and the transition temperature is defined as the middle point of the fitting curve, i.e. $T_{\mathrm{N}}$ = 27.1 (1) K, which is in excellent agreement with the $T_{\mathrm{N}}$ determined by magnetisation measurement (Fig. 3).

Below 20 K down to 2 K, the oscillation from the externally applied field is still clearly observed (see black curve in Fig. 4a), having a volume fraction of about 28$\%$. This suggests the existence of a second PM phase even at $T=2$ K. The absence of any detectable major impurity phases from diffraction measurements implies that the crystal structure of the second phase is the same (or very similar) as that of the predominant phase. This could be related to the broadening observed in high-resolution NPD data due to the distribution of atoms in the structure. Such scenario could lead to atomic and magnetic order/disorder transitions at low-temperatures^21^. In the honeycomb structure family, frustration is known to cause partial magnetic disorder. This leads to a spin liquid or spin glass like ground states, which is often hidden behind a long-range magnetic ordering^22–24^. Here the $\mu ^+$SR technique is uniquely capable of detecting such mixed state, including its volume fractions. For instance, previous high-field $\mu ^+$SR experiments on the related compound ${\hbox {Cu}_2\hbox {IrO}_3}$ shows a mixing of the two magnetic phases with a combination of static ordering of Cu$^2+$ and Kitaev spin liquid of both Cu$^+$ and Ir$^4+$^22^. The wTF-study of ${\hbox {Cu}_2\hbox {IrO}_3}$ shows that the oscillation from the externally applied field is visible even at 0.2 K, which corresponds to the Kitaev spin liquid phase. Since both ${\hbox {K}_2\hbox {Ni}_2\hbox {TeO}_6}$ and ${\hbox {Cu}_2\hbox {IrO}_3}$ have the similar honeycomb lattice of magnetic atoms, we might expect a similar second Kitaev spin liquid phase also in honeycomb ${\hbox {K}_2\hbox {Ni}_2\hbox {TeO}_6}$. Moreover, such exotic states could also explain the small divergence between *zfc* and *fc* below $T_{\mathrm{N}}$, as observed by the DC magnetic susceptibility (Fig. 3). In the case of ${\hbox {K}_2\hbox {Ni}_2\hbox {TeO}_6}$, there is only one magnetic atom, Ni$^{+2}$, however, it is known that the series of the compound can have a slightly different stacking sequence^25^, which can create multiple local magnetic environments, very similar to the ${\hbox {Cu}_2\hbox {IrO}_3}$ case. This scenario is further supported by the fact that the K-ions are indeed dynamic at room temperature (see below). Further low-temperature $\mu ^+$SR studies using $^3$He or dilution cryostat and under high longitudinal-field (LF) will be needed to clarify the interesting magnetic ground state of ${\hbox {K}_2\hbox {Ni}_2\hbox {TeO}_6}$.

### Low-temperature ZF $\mu ^+$SR  measurements

**Figure 5:**
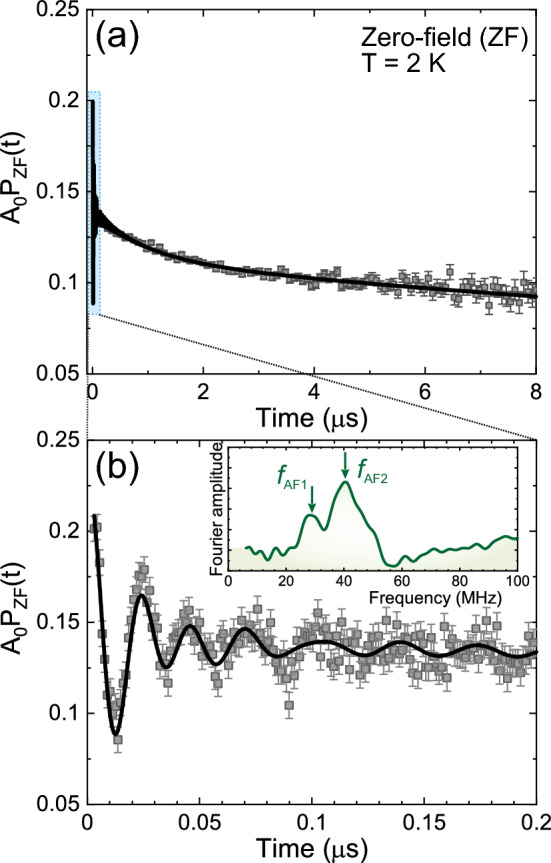
The ZF-$\mu ^+$SR time spectrum recorded at a temperature $T=2$ K under zero-field (ZF): (**a**) in the long time domain up to 8 $\upmu$s and (**b**) in an early time domain up to 0.2 $\upmu$s. The Solid lines are represent the best fits of the data using Eq. (2). The inset of (**b**) shows the Fourier transform (frequency spectrum) of the shorter time domain. Two frequencies ($f_{\mathrm{AF1}}$ = 29 MHz and $f_{\mathrm{AF2}}$ = 43 MHz) corresponding to the two (AF1 and AF2) oscillations are highlighted by black arrows.

To further understand the electronic spin order and hereby the magnetic nature of ${\hbox {K}_2\hbox {Ni}_2\hbox {TeO}_6}$, zero-field (ZF) $\mu ^+$SR  measurements were performed at temperatures between 2 and 40 K. As seen in Fig. 5, the ZF-$\mu ^+$SR time spectra recorded at 2 K clearly shows the muon spin precession signal, which evidences the appearance of quasi-static magnetic order. Fourier transform of the ZF-$\mu ^+$SR  time spectrum (inset of Fig. 5b) reveals the presence of two distinct components namely: $f_{\mathrm{AF1}}$ = 29 MHz and $f_{\mathrm{AF2}}$ = 43 MHz, with an asymmetry ratio of 1:10 (as is shown in Fig. 7d). In addition, there is a fast relaxing signal in the initial time spectra (see also Supplementary Fig. S1). Such behaviour may have several explanations; in particular this signal might be due to delocalised muons or fast fluctuating moments, arising from either the Ni ions or magnetic impurities. Thus, this ZF spectrum at 2 K was fitted by a combination of two exponentially relaxing cosine oscillations, which are originating from the magnetic order. One fast and one slow (for 1/3 powder average tail) exponentially relaxing non-oscillatory components and one exponentially relaxing non-oscillatory components due to the PM (or spin-liquid) signal observed in the wTF measurement ($A_{\mathrm{PM}}$ fixed at 0.0728). The resulting fit function is described as:

2 $$\begin{aligned} A_0 \, P_{\mathrm{ZF}}(t)= &\, {} A_{\mathrm{AF1}}\cos (2\pi f_{\mathrm{AF1}} t + {\phi _{\mathrm{AF1}}})\times {}\exp {(-\lambda _{\mathrm{AF1}} t)} \nonumber \\&+ A_{\mathrm{AF2}}\cos (2\pi f_{\mathrm{AF2}} t + {\phi _{\mathrm{AF2}}})\times {}\exp {(-\lambda _{\mathrm{AF2}} t)} \nonumber \\&+ A_{\mathrm{fast}}\times {}\exp {(-\lambda _{\mathrm{fast}} t)} \nonumber \\&+ A_{\mathrm{tail}}\times {}\exp {(-\lambda _{\mathrm{tail}} t)} \nonumber \\&+ A_{\mathrm{PM}}\times {}\exp {(-\lambda _{\mathrm{PM}} t)}, \end{aligned}$$

where $A_0$ is the initial asymmetry, $A_{\mathrm{AF1}}$, $A_{\mathrm{AF2}}$, $A_{\mathrm{fast}}$, $A_{\mathrm{tail}}$ and $A_{\mathrm{PM}}$ are the asymmetries associated with each signals, $f_{\mathrm{AF}}{_i}$ is the frequency of the muon spin precession corresponding to the static internal AF field, $\phi _{\mathrm{AF}}{_i}$ is the initial phase of the oscillatory signal, $\lambda _{\mathrm{AF}}{_i}$, $\lambda _{\mathrm{fast}}$, $\lambda _{\mathrm{tail}}$ and $\lambda _{\mathrm{PM}}$ are the exponential relaxation rates of each signal. $A_{\mathrm{PM}}$ was fixed at 0.0728, based on wTF measurements. As clearly shown in Fig. 5, the ZF-$\mu ^+$SR time spectrum is well fitted using Eq. (2) both in short (*t* $\le$ 0.2 $\upmu$s) and long (*t* < 8 $\upmu$s) time domain. Both $\phi _{\mathrm{AF1}}$ and $\phi _{\mathrm{AF2}}$ show similar temperature trend from individual fitting (not shown), thus a common $\phi _{\mathrm{AF}}$ was finally used in the fitting, i.e. $\phi _{\mathrm{AF}}$ = $\phi _{\mathrm{AF1}}$ = $\phi _{\mathrm{AF2}}$. Both $A_{\mathrm{AF1}}$ and $A_{\mathrm{AF2}}$ were also found to be almost temperature independent and were treated as common parameters in the temperature range between 2 and 23.5 K. The resulting values were obtained as $A_{\mathrm{AF1}}=0.0065$ and $A_{\mathrm{AF2}}=0.0811$ (Fig. 7d).

**Figure 6:**
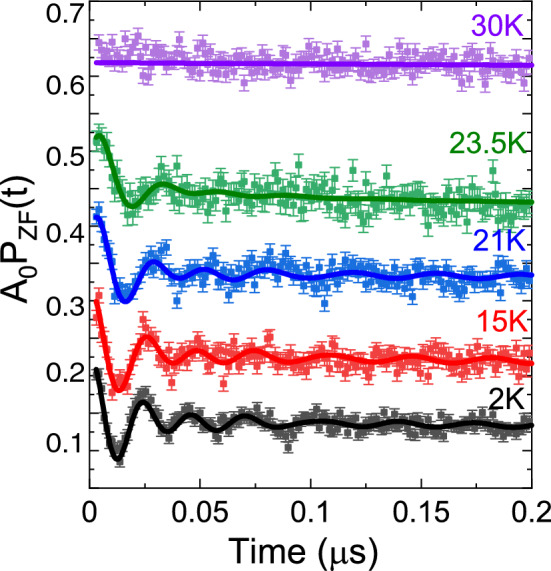
Temperature-dependent $\mu ^+$SR spectra for ${\hbox {K}_2\hbox {Ni}_2\hbox {TeO}_6}$ recorded under zero-field (ZF). Solid lines show the best fits with Eq. (2). Each spectrum is offset along the y-axis by 0.1, for clarity of display.

**Figure 7:**
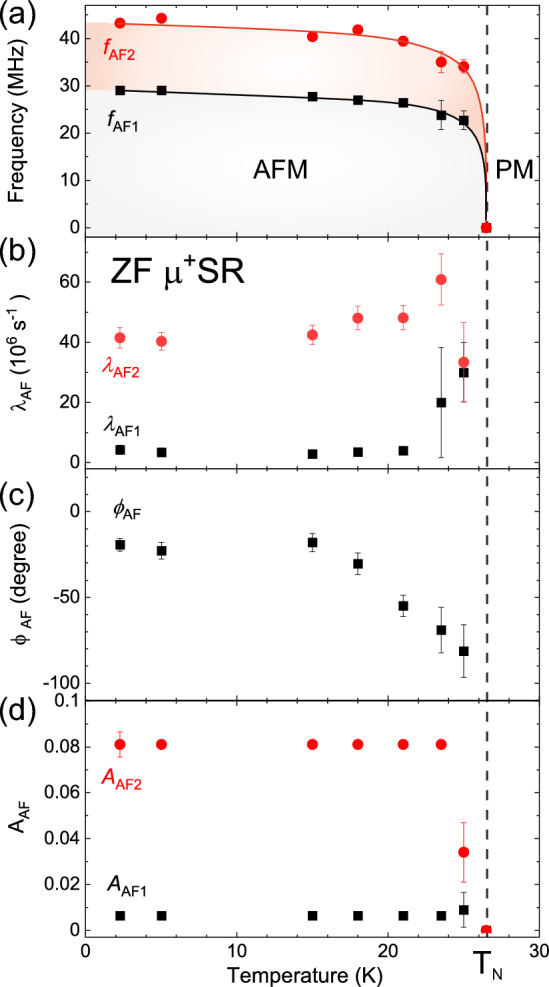
Temperature dependencies of the ZF-$\mu ^+$SR fitting parameters for ${\hbox {K}_2\hbox {Ni}_2\hbox {TeO}_6}$; (**a**) muon spin precession frequencies (f$_{\mathrm{AF1}}$ and f$_{\mathrm{AF2}}$), lines are guide to the eyes. (**b**) the relaxation rates ($\lambda _{AF}$), (**c**) the common initial phases of the two oscillatory signals ($\phi _{\mathrm{AF}}$) and (**d**) the asymmetries (A$_{\mathrm{AF}}$). The data were obtained by fitting the ZF-$\mu ^+$SR spectra using Eq. (2). Vertical dashed line indicates the antiferromagnetic transition temperature $T_{\mathrm{N}}\approx 27$ K determined by wTF-$\mu ^+$SR measurements.

Figure 6 shows the temperature dependence of ZF-$\mu ^+$SR time spectra [*t* < 0.2 $\upmu$s] recorded at temperatures between 2 and 30 K. The time spectra recorded below $T_{\mathrm{N}}$ (= 27 K) were well fitted using the Eq. (2) in both long and short time domains. Figure 7 shows the temperature dependence of the $\mu ^+$SR parameters obtained by fitting the ZF-$\mu ^+$SR spectrum with Eq. (2). As temperature decreases from 40 K, both $f_{\mathrm{AF1}}$ and $f_{\mathrm{AF2}}$ appears and drastically increase, reaching $\sim$ 75–93 $\%$ of its base temperature value already at $T=25$ K (Fig. 7a). Since $f_{\mathrm{AF}}$ corresponds to the order parameter of a magnetic transition, such a rapid AF transition is an indication of a first-order transition, which could be linked to a (multiple) structural phase transition. However, the co-existence of a structural and magnetic transition needs to be further investigated by low-temperature X-ray/neutron diffraction. Furthermore, these two frequencies seem to abruptly disappear almost at the same temperature $T_{\mathrm{N}}\approx 27$ K. This suggests that the two frequencies are not caused by the coexistence of two different phases in the sample but by two magnetically inequivalent muon stopping sites in the lattice. Further, although both $\lambda _{\mathrm{AF1}}$ and $\lambda _{\mathrm{AF2}}$ are roughly temperature independent below 20 K, $\lambda _{\mathrm{AF2}}$ increases with temperature below the vicinity of $T_{\mathrm{N}}$ (see Fig. 7b), indicating the increase of field (electronic spin) fluctuations close to $T_{\mathrm{N}}$.

The phase of the spin precession, $\phi _{\mathrm{AF}}$, is almost constant below 18 K, i.e. $\phi _{\mathrm{AF}}$ $\sim$ -20$^{\circ }$, while the magnitude of $\phi _{\mathrm{AF}}$ increases with temperature above 18 K (Fig. 7c). This suggests that the spin structure is most likely commensurate (C) to the crystal lattice. This is because an incommensurate (IC) AF structure usually provides a much large phase delay for a cosine function, typically $-45$ to $-60^{\circ }$, due to the mismatch between the IC magnetic modulation and muon sites. Indeed, usually a commensurate magnetic ordering gives $\phi _{\mathrm{AF}}\approx 0$. The observed small phase delay ($-20^{\circ }$) could instead be related to an artificial effect from the fit of very initial time domain for the fast oscillation. It could also be an effect from multiple muon stopping sites^26,27^. As an conclusion, the small delay of the initial phase is likely to support commensurate AF order in ${\hbox {K}_2\hbox {Ni}_2\hbox {TeO}_6}$, however, we would need further low-temperature neutron experiment to robustly confirm this.

Finally, all the $\mu ^+$SR parameters under ZF show a monotonic change in the temperature range between 2 K and $T_{\mathrm{N}}$. The present results hence suggest the absence of an additional magnetic transition down to 2 K, which is in good agreement with the magnetisation and wTF-$\mu ^+$SR results. Additional neutron diffraction studies at low-temperature would be the next future and natural step to shed further light on the magnetic nature of ${\hbox {K}_2\hbox {Ni}_2\hbox {TeO}_6}$.

### High-temperature K-ion diffusive properties

To study the solid-state K-ion diffusive properties of ${\hbox {K}_2\hbox {Ni}_2\hbox {TeO}_6}$, $\mu ^+$SR measurements above the magnetic transition temperature were performed. While the studies of the magnetically ordered state focused on the electronic spins of the TMO layers, the investigation of ion dynamics instead targets the nuclear moments of the potassium layers. Both Li-ion^26,28,29^ and Na-ion^30,31^ diffusive properties as a function of temperature have already been extensively studied using a series of ZF, wTF and LF-$\mu ^+$SR time spectra measurements, where LF means that the applied field is parallel to the initial muon spin polarization. However, since the nuclear magnetic moment of K ($\mu$[$^{39}$K] = 0.39 $\mu _{\mathrm{N}}$) is much smaller than that of Li ($\mu$[$^7$Li] = 3.26 $\mu _{\mathrm{N}}$) and Na ($\mu$[$^{23}$Na] = 2.22 $\mu _{\mathrm{N}}$), the measurement of K-ion dynamics using microscopic magnetic techniques^32,33^ is challenging. This means that $\mu ^+$SR could provide unique information on the K-ion diffusive properties, through its high sensitivity to local nuclear magnetic environments.

**Figure 8:**
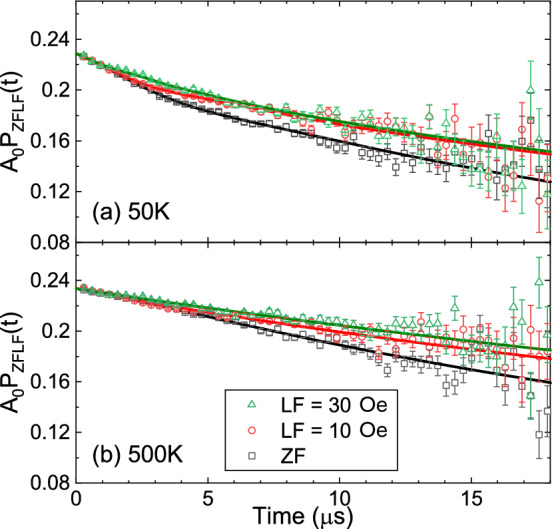
ZF and two LF (10 and 30 Oe) $\mu ^+$SR time spectra measured at (**a**) 50 K and (**b**) 500 K. Solid lines represent the fit result using Eq. (3).

To extract the onset and evolution of K-ion dynamics, $\mu ^+$SR time spectra were collected in the temperature range between 50 and 550 K using the EMU instrument of ISIS in UK. Figure 8 shows the ZF- and LF-$\mu ^+$SR time spectrum obtained at 50 K and 500 K. A decoupling behaviour by the applied LF (= 10 and 30 Oe), i.e. an *induced* reduction in the relaxation rate, is clearly visible even for small fields at both temperatures. This suggests that $H_{\mathrm{int}}$ sensed by the muons is mainly formed by nuclear magnetic moments. The small nuclear moment of each element (K, Ni, Te and O) in the compound yields a small field distributions width at the muon sites (i.e. small $\Delta$), resulting in what almost looks like an exponentially relaxing spectrum (while it is in fact a Kubo-Toyabe type spectrum^34^). This is also why it is essential to conduct these measurements at a pulsed muon facility that gives access to a longer time domain, and thereby yields a more robust fit to the data.

At each temperature, the ZF and the two LF spectra are found to be well fitted by a combination of two dynamic Gaussian Kubo-Toyabe (KT) functions, each multiplied by a simple exponential relaxation. The latter is due to a weak electronic relaxation related to the very fast fluctuating Ni spins. In addition, there is a small non-relaxing background (BG) signal from the fraction of muons stopped mainly in the silver plate mask on the sample holder. The resulting fit function for the ZF and two LF spectra is as follows:

3 $$\begin{aligned} A_0 \, P_{\mathrm{LF}}(t)= &\, {} A_{\mathrm{KT1}}G^{\mathrm{DGKT}}(\Delta _1, v_1, t, H_{\mathrm{LF}}) \times \exp ({-\lambda _{\mathrm{KT1}}t}) \nonumber \\&+ A_{\mathrm{KT2}}G^{\mathrm{DGKT}}(\Delta _2, v_2, t, H_{\mathrm{LF}}) \times \exp ({-\lambda _{\mathrm{KT2}}t}) \nonumber \\&+ A_{\mathrm{BG}}, \end{aligned}$$

Here $A_0$ is the total initial asymmetry, $A_{\mathrm{KT1}}$, $A_{\mathrm{KT2}}$ and $A_{\mathrm{BG}}$ are the asymmetries associated with each of the three components, $\Delta _1$ and $\Delta _2$ are related with the width of the local (nuclear) field distributions at the muon sites, $\nu _1$ and $\nu _2$ are the field fluctuation rates, and finally $\lambda _{\mathrm{KT1}}$ along with $\lambda _{\mathrm{KT2}}$ are the (electronic) relaxation rates. When $\nu$ = 0 and $H_{\mathrm{LF1}}$ = 0, $G^{\mathrm{DGKT}}(\Delta _1, v_1, t, H_{\mathrm{LF1}})$ becomes the simple static Gaussian KT function in ZF.

Furthermore, a fitting procedure with a common temperature independent background asymmetry ($A_{\mathrm{BG}}$ $\sim$ 0.04723) was employed, but with temperature dependent KT parameters, $\nu _1$, $\nu _1$, $\lambda _{\mathrm{KT1}}$, and $\lambda _{\mathrm{KT2}}$ (see also Supplementary Fig. S2). The two $\Delta$ were from individual fits found to be virtually temperature independent, and were therefore also treated as a common parameters over the entire temperature range [$\Delta _1$ $\sim$ 0.291 (23) $\upmu$s $^{-1}$ and $\Delta _2$ $\sim$ 0.043 (9) $\upmu$s $^{-1}$]. Thus, $\Delta _1$ is found to be close to an order of magnitude larger than $\Delta _2$. This is rather surprising if we would assume that the KT1 and KT2 components are related to the two muon stopping sites found at low temperature. This observation is further discussed and explained below.

**Figure 9:**
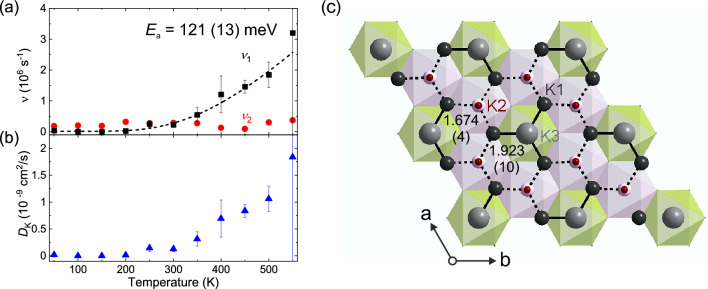
The temperature dependencies of (**a**) $\nu$ and (**b**) diffusion coefficient for ${\hbox {K}_2\hbox {Ni}_2\hbox {TeO}_6}$. The black dashed line is fit to an Arrhenius equation $\nu$ = $A \times \mathrm{exp}(-E_{\mathrm{a}}/k_{\mathrm{B}}T)$, which yield an activation energy of $E_{\mathrm{a}}$ = 121 (13) meV. Each data point was obtained by fitting the ZF and LF (= 10 and 30 Oe) spectra using Eq. (3). (**c**) Crystal structure of ${\hbox {K}_2\hbox {Ni}_2\hbox {TeO}_6}$ projection along *c*-axis. Diffusion paths, K1–K2 ($s_1$ = 1.674 Å) and K1–K3 ($s_2$ = 1.923 Å), are illustrated by dot line and solid line, respectively. Figure of crystal structure was created by Diamond version 4.6.3.

Figure 9a shows the temperature dependencies of $\nu _1$ and $\nu _2$, as extracted from fits of the $\mu ^+$SR data to Eq. (3). $\nu _2$ is almost constant over the whole temperature range ($\nu _2$ at 50 K is 0.184 (31)), while $\nu _1$ is close to zero up to 200 K, after which it clearly starts to increase. The exponential increase of $\nu _1$ between 200 and 550 K is typical for a thermally activated process, which signals the onset of diffusive motion of either ${\hbox {K}^+}$ or $\mu ^+$ above 200 K. Here, the scenario of K-ion diffusion is strongly supported by electrochemical investigations that clearly indicate that the K-ions are mobile in this temperature range^1,35^. Assigning the field fluctuation rate as the K-ion hopping rate in ${\hbox {K}_2\hbox {Ni}_2\hbox {TeO}_6}$, $\nu _1$(*T*) data is naturally fitted by an Arrhenius type equation (dashed line in Fig. 9a). Such fit provides the activation energy for the K-ion diffusion as $E_{\mathrm{a}}$ = 121 (13) meV. This value is comparable to the activation energy obtained by $\mu ^+$SR for Li based battery cathode materials, e.g. $E_{\mathrm{a}}=96$ meV for ${\hbox {Li}_{0.53}\hbox {CoO}_2}$^28^, $E_{\mathrm{a}}=124$ meV for ${\hbox {Li}_{0.98}\hbox {Ni}_{1.02}\hbox {O}_2}$^36^. Moreover, the temperature dependence of $\lambda _{\mathrm{KT2}}$ (Supplementary Fig. S2) is rather constant over the measured temperature while $\lambda _{\mathrm{KT1}}$ starts to decrease around room-temperature, and finally convergences to zero above 450 K. Here, the onset of K-ion dynamics revealed by $\nu _1(T)$ is clearly driving the change in $\lambda _{\mathrm{KT1}}(T)$, i.e. the same component (volume fraction). Details related to this observation are further discussed below.

From the absolute values of the ion hopping rate, we are then able to calculate the diffusion coefficient of the K-ions ($D_{\mathrm{K}}$). This procedure has previously been extensively used to determine the diffusion coefficient for Li and Na compounds^28,31^. The principle of diffusion of K$^+$ should be naturally the same to those for Li$^+$ and Na$^+$. Consequently, $D_{\mathrm{K}}$ is estimated via the following equation^37^:

4 $$\begin{aligned} D_{\mathrm{K}}= & {} \sum _{i=1}^{h} \frac{1}{N_i} Z_{\mathrm{v,i}} s_i^2 \nu , \end{aligned}$$

where $N_i$ is the number of possible K sites for the *i*-th jump path, $Z_{\mathrm{v,i}}$ is the vacancy fraction and $s_i$ is the jump distance for such path. Naturally, we restrict the diffusion path within the 2D potassium layer of the honeycomb. Moreover, we assume a diffusion path only within the nearest neighbour sites within the honeycomb flower as shown in Fig. 9c where only two K-diffusion pathways are allowed, that is, K1–K2 and K1–K3. The values for *s* and *Z* are extracted from our neutron diffraction measurements [see also the refined structural parameters in Supplementary Tables S1 and S2], Since *s* directly relates to the inter atomic distances of potassium, $s_1=1.674$ Å for K1–K2 ($N_1=5$) and $s_2=1.923$ Å for K1–K3 ($N_2= 4$). Based on such assumption, we obtain $D_{\mathrm{K}}^{\mathrm{300~K}}=0.13\times 10^{-9}$ cm$^2$/s using $\nu _1$(300 K) = 0.29 $\upmu$s$^{-1}$. This value is one order of magnitude lower than $D_{\mathrm{Li}}^{\mathrm{300~K}}$ for the archetypical Li-ion battery cathode material ${\hbox {LiCoO}_2}$^28^. $D_{\mathrm{K}}$ for ${\hbox {K}_2\hbox {Ni}_2\hbox {TeO}_6}$ are also calculated for the other temperatures as shown in Fig. 9b, e.g. $D_{\mathrm{K}}^{\mathrm{400~K}}= 0.69\times 10^{-9}$ cm$^2$/s using $\nu _1$(400 K) = 1.21 $\upmu$s$^{-1}$ and $D_{\mathrm{K}}^{\mathrm{500~K}}=1.06\times 10^{-9}$ cm$^2$/s using $\nu _1$(500 K) = 1.85 $\upmu$s$^{-1}$. Here we have assumed that the atomic structure remains the same within the entire temperature range. To further investigate the ion diffusion in ${\hbox {K}_2\hbox {Ni}_2\hbox {TeO}_6}$, detailed studies of the temperature dependency of the atomic structure using X-ray and/or neutron diffraction would be useful. Such investigations could yield even more accurate information on the active diffusion pathways^38^, which would allow us to further refine the calculations of $D_{\mathrm{K}}$ from $\nu (T)$, especially as a function of temperature.

## Discussion

Concerning the two KT components used in the fit function of the ion diffusion measurements at higher temperature. It should be noted that the KT1 signal that reveals the strong temperature dependence in K-ion hopping rate ($\nu _{1}$) constitutes the minor volume fraction (asymmetry). This could be due to that the two different muon stopping sites are very different in relation to the K-ion layers, and that KT2 is related to a site where the muon is screened from detecting dynamic changes in the weak nuclear moment of potassium. Such scenario is supported by the fact that in the low-temperature $\mu ^+$SR data the larger volume fraction relates to the higher frequency ($f_{\mathrm{AF2}}$), which indicate that such muon site is located closer to the TMO layer. However, it is questionable that it would be possible to distinguish the separate contributions (in the fitting) of two muon sites in the paramagnetic state. Another, in our opinion more probable scenario, is that KT1 and KT2 relates to surface and bulk signals, respectively. Such interpretation is supported by the temperature dependence of $\lambda _{\mathrm{KT1}}$ and $\lambda _{\mathrm{KT2}}$ (Supplementary Fig. S2b) that display very different behaviour. Such data is coherent with our previous work on the well-known ${\hbox {LiFePO}_4}$ cathode material^26,29,39,40^, where we indeed have shown by both inelastic neutron scattering and $\mu ^+$SR that the self-diffusion of lithium ions is mainly limited to the surface region of the ${\hbox {LiFePO}_4}$ particles. Our current results indicate that the situation could be very similar also for ${\hbox {K}_2\hbox {Ni}_2\hbox {TeO}_6}$. From the temperature average of the asymmetries ($A_{\mathrm{KT1}}$ and $A_{\mathrm{KT2}}$) it is found that the volume fraction of the supposed surface region that display K-ion diffusion is about 8%. It is known that the size of the ${\hbox {K}_2\hbox {Ni}_2\hbox {TeO}_6}$ powder particles are approximately 300–350 nm (see supplementary material of Ref.^1^). For simplicity if we consider fully spherical particles, an asymmetry volume fraction of 8% would correspond to an active surface layer that is approximately 4 nm thick. This is very reasonable and indeed a very important information for the future application of this material in battery devices. This also clearly show the power of the $\mu ^+$SR technique for studying energy related materials. This is the only technique available that can directly and locally probe the volume fraction of ion diffusion in bulk materials. This allow us to uniquely study important surface and interface properties in, e.g. battery materials. This can be conducted either indirectly via bulk $\mu ^+$SR techniques, like our current and previous studies^40^, or by the utilization of the low-energy $\mu ^+$SR (LEM) method that is able to directly probe the surface/interface properties via depth-resolved studies of thin-film and multi-layer samples^41^. To conclusively confirm the origin of the two KT functions as surface and bulk contributions, further theoretical calculations to robustly determine the muon sites along with additional systematic $\mu ^+$SR and potentially LEM studies of nano-structured samples with controllable size and surface will be required. Such investigations would also clarify what the physical difference is between the surface and bulk regions, e.g. local structure/disorder, stacking sequence, K-ion vacancies/occupancy, etc.

In conclusion, muon spin rotation and relaxation ($\mu ^+$SR) together with bulk magnetization measurements of ${\hbox {K}_2\hbox {Ni}_2\hbox {TeO}_6}$ reveal the formation of a commensurate-like antiferromagnetic order at $T_{\mathrm{N}}\approx 27$ K. Further, potassium-ions (K$^+$) in ${\hbox {K}_2\hbox {Ni}_2\hbox {TeO}_6}$ are found to be mobile above $T=200$ K, with remarkably low activation energy, $E_{\mathrm{a}}=121 (13)$ meV. This is comparable to the thermal activation energy scales of related lithium- and sodium-based materials. Moreover, assisted by high-resolution neutron diffraction measurements, we are also able to estimate the local self-diffusion coefficient of K-ion ($D_{\mathrm{K}}$) as a function of temperature. This brings related honeycomb layered oxide materials to the foreground of fast ionic conductors for energy storage. With these results, we have shown, for the first time, the feasibility of the $\mu ^+$SR technique for investigating ion (K$^+$) dynamics in materials containing low nuclear magnetic moments. This study expands the research frontier of alkali-ion dynamics in energy materials, previously limited to mainly lithium and sodium compounds. Finally our results also reveal that K-ion self diffusion in ${\hbox {K}_2\hbox {Ni}_2\hbox {TeO}_6}$ is highly governed by an approximately 4 nm thin surface region of the powder particles. This important result opens future possibilities for improving ion diffusion and K-ion battery device performance by nano-structuring and/or surface treatments of the particles.

## Experimental section

### Materials synthesis

Polycrystalline powder of ${\hbox {K}_2\hbox {Ni}_2\hbox {TeO}_6}$, (${\hbox {K}_{2/3}\hbox {Ni}_{2/3}\hbox {Te}_{1/3}\hbox {O}_2}$) was synthesised using a high-temperature ceramics route. Stoichiometric amounts of NiO [99.9 $\%$ purity, Kojundo Chemical Laboratory (Japan)], ${\hbox {TeO}_2}$ (99.0 $\%$ purity, Aldrich) and ${\hbox {K}_2\hbox {CO}_3}$ [99.9$\%$ purity, Rare Metallic (Japan)] were mixed, pressed into pellets and finally heated for 23 h at 800$^{\circ }$C in air. The obtained powders were stored in an argon-purged glove box that was maintained at a dew point of below − 80$^{\circ }$C dP, to prevent exposure of the materials to moisture. More detailed information on the synthesis protocol can be found in Ref.^1^.

### X-ray and neutron powder diffraction

Sample quality was checked by room-temperature X-ray powder diffraction (Cu-K$\alpha$ radiation). Room-temperature neutron powder diffraction was performed on the high-resolution time-of-flight SPICA beamline at J-PARC/MLF in Japan^42^. Structural refinements were performed with the FullProf suite of programs^43^, taking into account anisotropic strains using a spherical harmonics modelling of the Bragg peak broadening. For the diffraction experiments the samples were carefully packed and sealed inside the Vanadium sample container using a glove-box in order to avoid sample degradation or contamination.

### Magnetic susceptibility measurements

Magnetic measurements as a function of temperature were performed with a 9 T Quantum Design superconducting quantum interference device (SQUID) magnetometer in zero-field-cooled (*zfc*) and field-cooled (*fc*) modes. Data from both modes were collected upon warming the sample *T* =5–300 K. The magnetic susceptibility ($\chi$) was obtained using the equation $\chi$ = *M*/*H*, where *M* is the magnetisation obtained by dividing the measured magnetic moment by the sample mass and *H* is the external applied magnetic field (in Oe).

### Muon spin rotation and relaxation ($\mu ^+$SR) measurements

**Figure 10:**
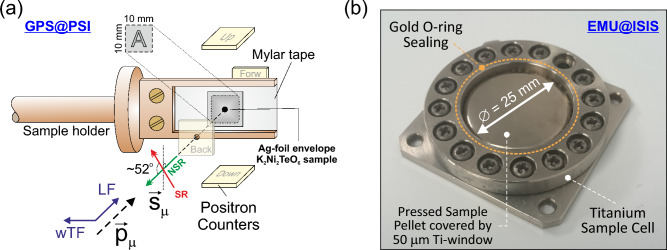
(**a**) Illustration of the experimental setup used to perform muon spin rotation and relaxation ($\mu ^+$SR) measurements at the GPS/PSI spectrometer. The muons are implanted into the sample through the back detector, where $\vec {P}_{\mu }$ is the momentum vector of the muon and $\vec {S}_{\mu }$ the spin vector pointing away from the direction of motion. The muons stops inside the sample and subsequently decay into positrons, which are counted by the detectors, and neutrinos. LF denotes the longitudinal field whereas wTF is the weak transverse field applied. (**b**) Sealed and mounted titanium sample cell used for the $\mu ^+$SR ion diffusion measurements at EMU/ISIS. Figures were created by using CorelDRAW 2019.

$\mu ^+$SR experiments were in similarity to our previous studies of low-temperature magnetic properties^27,44,45^ performed using a positive surface muon beam line and the GPS spectrometer at the Swiss Muon Source (S$\mu$S in PSI, Switzerland). The handling of the powder sample ($m\approx 0.5$ g) was performed inside a glove-box (controlled He(g) atmosphere) to avoid sample degradation due to mainly humidity. The sample container was made out of very thin folded silver foil (25 $\upmu$m) sealed by low vapour pressure epoxy glue (Torr Seal). The final sample *envelope* had a $10\times 10$ mm$^2$ surface area and was about 1 mm thick. The sample was was attached to a fork-type (low-background) sample holder made of non-magnetic oxygen-free high thermal conductivity (OFHC) copper (see Fig. 10a) using a single layer of Al-coated Mylar tape. The sample holder was affixed to a stick and inserted into the GPS instrument cryostat (liquid-He flow-type) for measurements in the temperature range *T* = 2–50 K. For each temperature $\mu ^+$SR time spectra were collected using both weak transverse-field (wTF = 20 Oe) and zero-field (ZF) protocols. Here the wTF measurements are performed in order to directly extract the magnetic volume fraction of the sample, but also to obtain a first overview of the temperature dependence. The more time demanding ZF measurements are thereafter used to more carefully extract the details of the intrinsic magnetic spin order and dynamics at selected temperatures under zero applied external field.

For the high-temperature ion diffusion measurements, $\mu ^+$SR time spectra were recorded using the EMU spectrometer at the pulsed muon source of ISIS/RAL in UK. A powder sample of ${\hbox {K}_2\hbox {Ni}_2\hbox {TeO}_6}$ ($m\approx$ 1 g) was pressed into a pellet with a diameter and thickness of 25 mm and 3.0 mm, respectively. This pellet was packed into a sealed (gold O-ring) powder cell made of non-magnetic titanium using a thin (50 $\upmu$m) Ti-film window (see Fig. 10b). The sample preparation was performed inside a helium glove-box to avoid sample degradation. In addition, a silver mask was mounted onto the Ti-cell to ensure that any (minor) background signal would be non-relaxing over a wide temperature range. The cell was mounted onto a Cu end-plate of the closed-cycle refrigerator (CCR) and measurements were performed at temperatures between 50 and 550 K. $\mu ^+$SR time spectra were subsequently collected using ZF, wTF = 20 Oe and longitudinal-field (LF = 10 and 30 Oe) protocols. Prior to the sample studies, systematic calibration measurements were conducted using the same titanium sample cells and silver mask. By using an Ag-plate instead of the sample, the maximum initial asymmetry for the setup is extracted. Replacement with a hematite pellet, the volume fraction of the background signal originating from the silver mask is extracted. Such calibration data were then used as input and boundary conditions for some of the fitting parameters during the analysis of data collected during the actual sample investigations.

Further details regarding the experimental techniques and set-ups are provided in Fig. 10 as well as in Ref.^46^. The musrfit^47^ software package was used to analyse the $\mu ^+$SR data both from both GPS and EMU studies.

## Supplementary information

Supplementary Information
