# Supplementary material for: Magnetism and ion diffusion in honeycomb layered oxide K2Ni2TeO6
Source: Sci Rep. 2020 Oct 27;10:18305. doi: 10.1038/s41598-020-75251-x (PMC7591923; doi:10.1038/s41598-020-75251-x)
Supplement: Supplementary file 1 — Supplementary Information [file 41598_2020_75251_MOESM1_ESM.pdf]

Supporting Information for: Magnetism and Ion Diffusion in Honeycomb Layered Oxide $K_2Ni_2TeO_6$

Nami Matsubara[1,*], Elisabetta Nocerino[1], Ola Kenji Forslund[1], Anton Zubayer[1], Konstantinos Papadopoulos[2], Daniel Andreica[3], Jun Sugiyama[4], Rasmus Palm[1], Zurab Guguchia[5], Stephen P. Cottrell[6], Takashi Kamiyama[7], Takashi Saito[7], Alexei Kalaboukhov[8], Yasmine Sassa[2], Titus Masese[9,10], and Martin Månsson[1,+]

[1]Department of Applied Physics, KTH Royal Institute of Technology, SE-10691 Stockholm, Sweden
[2]Department of Physics, Chalmers University of Technology, SE-41296 Gothenburg, Sweden
[3]Faculty of Physics, Babes-Bolyai University, 400084 Cluj-Napoca, Romania
[4]Neutron Science and Technology Center, Comprehensive Research Organization for Science and Society (CROSS), Tokai, Ibaraki 319-1106, Japan
[5]Laboratory for Muon Spin Spectroscopy, Paul Scherrer Institute, CH-5232 Villigen PSI, Switzerland
[6]ISIS Muon Facility, Rutherford Appleton Laboratory, Didcot, Oxfordshire, OX11 0QX, UK
[7]Institute of Materials Structure Science, High Energy Accelerator Research Organization, 203-1 Shirakata, Tokai, Ibaraki 319-1107, Japan
[8]Microtechnology and Nanoscience, Chalmers University of Technology, SE-41296 Gothenburg, Sweden
[9]Department of Energy and Environment, Research Institute of Electrochemical Energy (RIECEN), National Institute of Advanced Industrial Science and Technology (AIST), Ikeda, Osaka 563-8577, Japan
[10]AIST-Kyoto University Chemical Energy Materials Open Innovation Laboratory (ChEM-OIL), National Institute of Advanced Industrial Science and Technology (AIST), Sakyo-ku, Kyoto 606-8501, Japan
[*]namim@kth.se
[+]condmat@kth.se

Table 1. Structural parameters of $K_2Ni_2TeO_6$ at 300 K from Reitveld refinements of XRPD and NPD data. The space group is $P6_3/mcm$. Te atoms are in the (2*b*) Wyckoff position (WP) [0 0 0], Ni in (4*d*) [2/3 1/3 0], O in (12*k*) [*xxz*], K1 in (6*g*)[x 0 1/4], K2 in (4*c*) [1/3 2/3 1/4] and K3 in (2*a*) [0 0 1/4]. The refined *x* and *z* parameters and isotropic thermal factors (*B*) are given for the different ions. The occupancy of K sites are also refined (*Oc*). Maximum values among the refinement results of different banks (global fit)R_{Bragg} is described here.

		NPD
	a (Å)	5.258 (1)
	c (Å)	12.42 (1)
	V (Å^3)	297.3 (1)
Te	B (Å^2)	0.33 (10)
Ni	B (Å^2)	0.45 (3)
O	x	0.6887 (3)
	z	0.5843 (1)
	B (Å^2)	0.47 (2)
K1	x	0.3657 (19)
	B (Å^2)	3.89 (33)
	Oc	0.494 (1)
K2	B (Å^2)	3.89 (33)
	Oc	0.272 (1)
K3	B (Å^2)	3.89 (33)
	Oc	0.020(1)
	R_{Bragg} (%)	6.56

Table 2. Selected distances (Å) of $K_2Ni_2TeO_6$ obtained from XRPD and NPD data

	NPD
Te - O	1.943 (2)
Ni - O	2.094 (2)
K1 - K2	1.674 (4)
K1 - K3	1.923 (10)
Ni - Ni	3.036 (1)

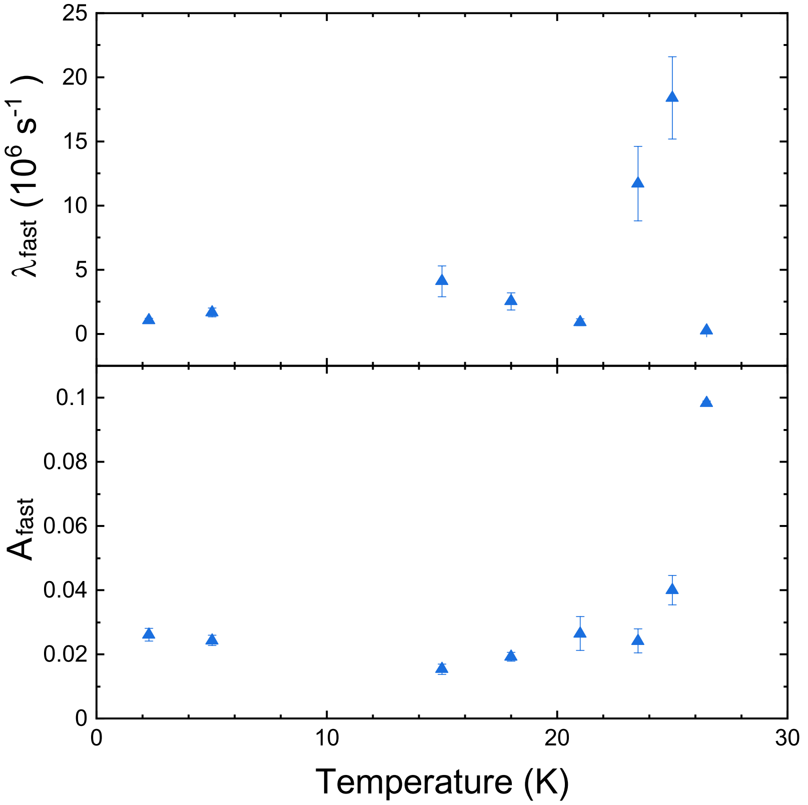

Figure 1. Temperature dependencies of μ^+SR parameters taken for $K_2Ni_2TeO_6$; (top) the relaxation rates (λ_{fast}) and (bottom) the asymmetries (A_{fast}). The data were obtained by fitting the ZF spectra using Eq.2 described in the main text.

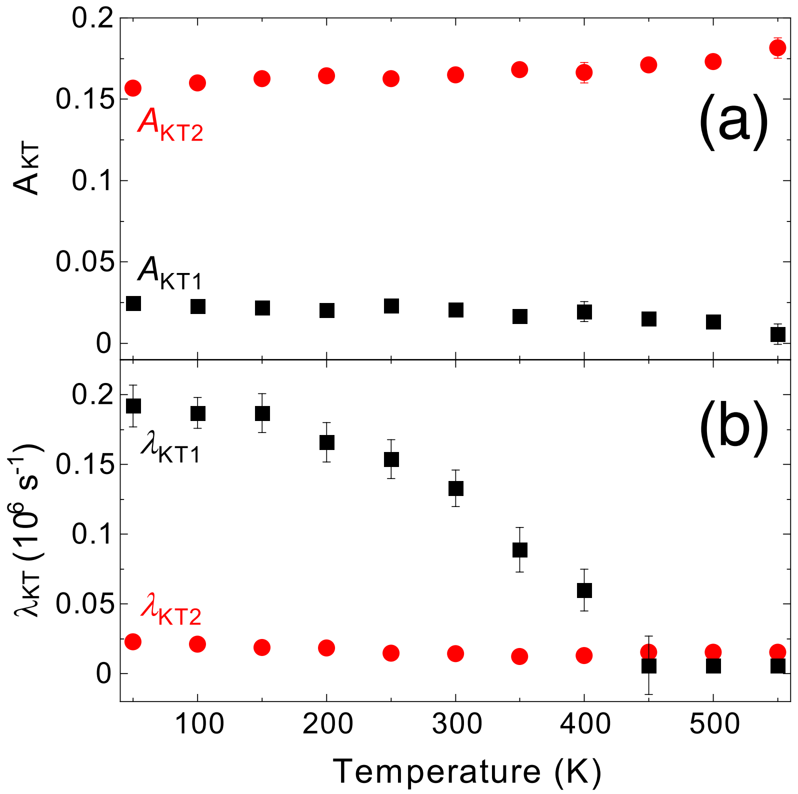

Figure 2. The temperature dependencies of (a) the asymmetry (A_{KT}) and (b) λ_{KT} for $K_2Ni_2TeO_6$.
